# ïSCOPE: Safer care for older persons (in residential) environments: A study protocol

**DOI:** 10.1186/1748-5908-6-71

**Published:** 2011-07-11

**Authors:** Lisa A Cranley, Peter G Norton, Greta G Cummings, Debbie Barnard, Carole A Estabrooks

**Affiliations:** 1 Faculty of Nursing, University of Alberta, Edmonton, Alberta, Canada; 2 Department of Family Medicine, University of Calgary, Calgary, Alberta, Canada

## Abstract

**Background:**

The current profile of residents living in Canadian nursing homes includes elder persons with complex physical and social needs. High resident acuity can result in increased staff workload and decreased quality of work life.

**Aims:**

Safer Care for Older Persons [in residential] Environments is a two year (2010 to 2012) proof-of-principle pilot study conducted in seven nursing homes in western Canada. The purpose of the study is to evaluate the feasibility of engaging front line staff to use quality improvement methods to integrate best practices into resident care. The goals of the study are to improve the quality of work life for staff, in particular healthcare aides, and to improve residents' quality of life.

**Methods/design:**

The study has parallel research and quality improvement intervention arms. It includes an education and support intervention for direct caregivers to improve the safety and quality of their care delivery. We hypothesize that this intervention will improve not only the care provided to residents but also the quality of work life for healthcare aides. The study employs tools adapted from the Institute for Healthcare Improvement's Breakthrough Series: Collaborative Model and Canada's Safer Healthcare Now! improvement campaign. Local improvement teams in each nursing home (1 to 2 per facility) are led by healthcare aides (non-regulated caregivers) and focus on the management of specific areas of resident care. Critical elements of the program include local measurement, virtual and face-to-face learning sessions involving change management, quality improvement methods and clinical expertise, ongoing virtual and in person support, and networking.

**Discussion:**

There are two sustainability challenges in this study: ongoing staff and leadership engagement, and organizational infrastructure. Addressing these challenges will require strategic planning with input from key stakeholders for sustaining quality improvement initiatives in the long-term care sector.

## Background

Approximately 70% of people with dementia will die in a residential long-term care (LTC) facility [1], commonly referred to as a nursing home. Almost one-half of Canadians in LTC facilities are frail elderly over 80 years of age [2,3]. Furthermore, present prevalence estimates indicate that the number of people with dementia in Canada will almost triple by 2038 to 1.25 million [4]. People with dementia have complex care needs and a high dependency on their providers, particularly during end-stage dementia. High resident acuity can result in increased staff workload and decreased quality of work life [5]. Several reports at international [6], national [7], and provincial levels [8] describe the sub-optimal quality of care in nursing homes. With people living longer and with the growing numbers of those living with dementia, the need for quality LTC for the elderly will continue to increase dramatically [9].

### Threats to quality and safety in care in nursing homes

Over the past decade, we have seen increasing efforts to develop and test methods to address quality of care and safety [10-13]. The Canadian Patient Safety Institute comprehensive plan focuses on strategies that will continually improve cultures of safety in healthcare to establish the safest health system for all Canadians [13]. Quality of work life in healthcare settings affects both patient outcomes and crucial staff outcomes such as retention [14,15]. The growing number of residents in nursing homes with dementia increases job strain [16] and job-related stress [17] of healthcare providers, leading to reduced job satisfaction [17] and ultimately staff turnover. High turnover has been linked to poor resident outcomes, such as decreased functional ability and pressure ulcers [18]. Staff turnover in nursing homes is higher than in many other types of organizations [19]. Healthcare aides (HCAs), who provide 70 to 80% of direct resident care, often leave nursing homes within months of employment [19].

Several studies have demonstrated that staff satisfaction and engagement are related to quality of care for residents of nursing homes [20-22]. Staff engagement is the involvement and commitment of staff [20,23] and 'a heightened emotional and intellectual connection that an employee has for his/her job, organization, manager, or co-workers that, in turn, influences him/her to apply additional discretionary effort to his/her work' [21]. There is evidence that teamwork contributes to performance by reducing errors and improving the quality of patient care [24]. Team performance has been associated with improved patient outcomes [25] and improved quality of care in LTC [26]. Yeatts *et al. *[26] reported that certified nursing assistant empowered work teams had modest positive effects on (improved) empowerment and performance, coordination and cooperation with nurses, and on residents' care. Others have suggested that improving communication and leadership among staff in nursing homes can facilitate team cohesion [27] and improve quality of care [28]. Interdisciplinary team functioning is particularly important in caring for frail elderly because of their complex needs, requiring effective coordination of resources [27]. Others have found that teams with a champion perceived themselves to be more effective [29].

### Study purpose and objectives

The purpose of the study, which is called Safer Care for Older Persons [in residential] Environments (SCOPE), is to evaluate the feasibility of an intervention designed to engage front line staff (primarily HCAs) in using quality improvement (QI) methods to integrate evidence-based (best) practices into resident care. The overall goals of this study are: to support HCAs in learning and using QI methods to improve safety and quality of care for the elderly living in nursing homes; and, through the resulting empowerment, improve the quality of work life for staff providing direct care in these nursing homes.

### Theoretical framing

The SCOPE study is guided by the Model for Improvement developed by Associates in Process Improvement [30]. The model has two parts:

1. Three fundamental questions, which can be addressed in any order:

a. What are we trying to accomplish?

b. How will we know that a change is an improvement?

c. What changes can we make that will result in improvement?

2. Changes are tested using the Plan-Do-Study-Act (PDSA) cycle of rapid change in real work settings [31]. The PDSA cycle guides the test of a change to determine if the change is an improvement [32].

The fundamental premise is that front line healthcare providers know their processes of care and can, using this simple change management system, improve these processes. The model enables staff to bring evidence-based care to the bedside.

### Design

**Figure 1:**
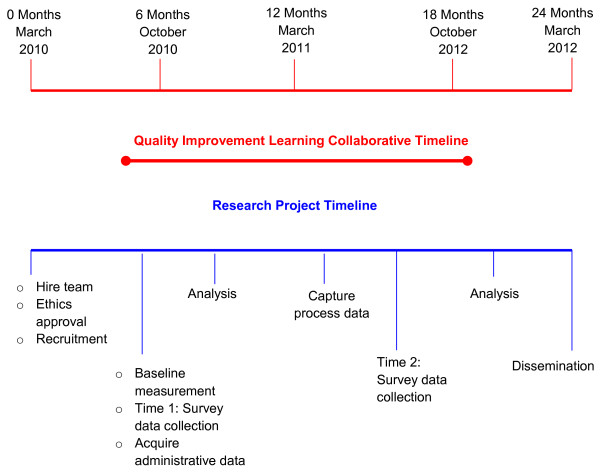
**Overview of research study arms**.

**Figure 2:**
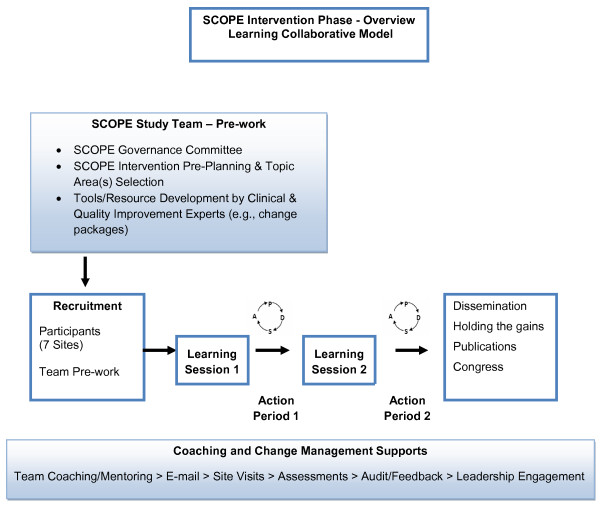
**Overview of SCOPE learning collaborative model**. Adapted from the Institute for Healthcare Improvement Breakthrough Series Collaborative [33].

This study is a two-year (2010 to 2012) proof of principle pilot that has research and QI intervention arms that run parallel (Figure 1). SCOPE is a 'bundle' of knowledge translation strategies designed to facilitate the successful implementation of changes at the clinical/unit level in selected clinical domains and to increase the engagement of front line staff in decision-making and action to improve practice and resident outcomes. The intervention is facilitation, coaching, and networking of QI teams. The intervention is designed on the Institute for Healthcare Improvement (IHI) Breakthrough Series Collaborative model [33]. The Breakthrough Series Collaborative is a shared learning system that brings together teams who seek improvement to work on focused topic areas with subject matter and QI experts [33]. The key components of the intervention are shown in Figure 2 and include: clinical and QI resources; face-to-face learning sessions, followed by two action periods where teams are coached virtually to test change ideas in their local environments; access to clinical and improvement experts; and support to track process measures (*e.g*., work group communication) and resident outcome measures (*i.e*., Resident Assessment Instrument - Minimum Data Set 2.0 or RAI-MDS 2.0). Table 1 shows key components of the intervention summarized in quality and knowledge translation language. The SCOPE Learning Collaborative has two face-to-face learning sessions and a closing congress to celebrate successes and develop strategies for spread and sustainability of QI work in the LTC sector. This learning collaborative also integrates learning and strategies used in the Canadian improvement campaign Safer Healthcare Now! primarily in acute care settings [34].

**Table 1:** SCOPE bundle of strategies

The SCOPE 'bundle' (framed in Quality language)	The SCOPE 'bundle' (framed in Knowledge Translation language)
1. Change packages	1. Evidence based practice and implementation strategies
2. Learning Sessions	2. Change management and measurement skills training and development
3. Action Periods	3. Testing change strategies
• PDSA: Plan-Do-Study-Act	• hypothesize - collect data-examine data against hypothesis - rethink hypothesis^1^
4. Coaching & Mentoring	4. Facilitation/support
• Monthly teleconferences	
• Emails	
• Project management system	
• Team reports	
• Senior Sponsor reports	
5. Monthly feedback reports	5. Monthly feedback reports

^1 ^http://www.improve.org.au/content/What_is_quality_improvement.html

## Methods

### Setting and facility sample

The study is being conducted in seven urban nursing homes--two in Alberta and five in British Columbia. Eligible facilities in each jurisdiction were identified with assistance from the study's decision makers. Facility selection was made using a convenience sample of nursing homes that met the inclusion criteria outlined in Table 2.

**Table 2:** Facility inclusion and exclusion criteria

Inclusion criteria	
1.	The facility is registered by the respective provincial governments

2.	The majority of residents are over 65 years of age

3.	The facility must have conducted RAI-MDS 2.0^1 ^assessment for at least one year and continue to collect these data

4.	The facility conducts operations in the English language

5.	Healthcare aides must provide greater than 50% of direct care

6.	The facility administrator (or region or owner-operator) is willing to sign a data sharing agreement

7.	A commitment from the facility administrator to have a senior sponsor (*e.g*., care manager, Director of Care) available to support the improvement team on a monthly basis

8.	A commitment from the facility administrator to release the equivalent of approximately 5 to 10% of a healthcare aide position for study related activities during the 12 months the intervention is implemented

9.	A commitment from the facility administrator to financially support staff team member attendance at the learning sessions (up to $3,000)

Exclusion criteria	

1.	The facility has a sub-acute unit

2.	The facility is integrated into an acute care facility

3.	The facility has less than 75 beds

^1^Resident Assessment Instrument-Minimum Data Set 2.0

### Quality improvement team sample

Administrators from the volunteering nursing homes are asked to identify a team of front line caregivers with the majority being HCAs. Each team is composed of four or five staff, including two or three HCAs and one or more registered professional staff (*e.g*., physiotherapist) who meet the following study inclusion criteria: work a minimum of six shifts per month; identify a unit where they work most of the time; and able to read and write English. Each team is led by a HCA and is supported by a local Senior Sponsor (*e.g*., care manager, director of care, vice-president) who serves effectively as a champion. HCA students were not eligible to participate in the QI teams because they are not directly affiliated with a nursing home. Research team members provide staff with an information letter about the study including purpose, activities, and time commitment involved with participating as a QI team member. Consent for participation in the QI teams is obtained either during the information session or in a subsequent visit to the nursing homes.

### Intervention procedure: The quality improvement arm

The intervention runs for 12 months (October 2010 to October 2011). Staff participating in the intervention (*e.g*., HCAs, nurses, physiotherapists) form QI teams to implement strategies to improve one of three possible areas of resident care: pain management, behaviour management, and skin care/pressure ulcer prevention and management. The selection of the area of focus is carried out locally by the teams. To predetermine the three areas we used a Delphi approach [35] to generate a short list of domains of resident care from the list of RAI-MDS 2.0 quality indicators [36]. Five stakeholder groups were solicited (email or face-to-face) to identify, prioritize, and seek consensus on RAI-MDS 2.0 quality indicators that are relevant and important to HCAs work: gerontology experts, senior decision makers, HCAs, registered nurses/care coordinators, and managers/educators. The top five priority areas of care for improvement are ranked, and QI teams with support from the QI advisor (from the SCOPE research team), care manager and senior sponsor at the nursing home are asked to identify one area of care from the list of five to work on improving as a team.

For each of the three topic areas we prepared a change package outlining current evidence, practical guidelines on how the evidence could be translated and implemented to direct resident care, the Improvement Model, and other basic QI methods. These were expanded upon at learning sessions which also provide opportunities for team members to: meet face-to-face and to practice QI techniques and strategies; receive individual coaching from clinical and improvement experts; gather new knowledge about their chosen topics; share new experiences and collaborate on improvement plans; and develop strategies to overcome barriers in their local environments. The learning sessions (1.5 days each) are held provincially (one in Alberta and one in British Columbia). A face-to-face team meeting is held in spring 2011 in each of the two participating provinces. Action periods between the learning sessions provide teams with time to test change strategies in their local settings. The overall aim of the action periods is for the teams to work on putting the 'best practices' included in the change package into practice. The key activities for action periods are carried out by teams with support from the QI advisor and senior sponsors including: setting aims, establishing measures, selecting changes, testing changes, measuring changes, and communicating shared learning [30].

### Feedback Reports

Teams are given feedback on their selected area of resident care. Reports are produced as run charts, and consist of data from RAI-MDS 2.0 and process data collected by teams. Teams can use the feedback to track their performance and progress towards their improvement goal. These reports assist teams to refine their change strategy if needed (*i.e*., act on what is learned).

### The research arm

The research arm uses a pretest-posttest design. We use the SCOPE survey (described in a later section) to gather data about organizational context, research use, and staff outcomes (*e.g*., job satisfaction) in all units in the nursing homes involved in the study.

All HCAs in each nursing home are invited to complete the SCOPE survey. The inclusion criteria for selecting HCAs to complete this survey are: employed by the facility for a minimum of three months, identify a unit where they work most of the time, and able to read and write English.

### Recruitment of HCA survey respondents

Research team members conduct short information sessions (10 to 15 minutes) with HCAs during scheduled times, facilitated by unit managers. A study flyer is posted in each participating nursing home. Staff are given an information letter about the study. Consent for participation in the survey is obtained from HCAs prior to completing the survey.

### HCA survey administration

We are conducting surveys with HCAs in the seven nursing homes before (Time 1) and after (Time 2) the QI intervention using a modified version of the survey used in the Translating Research in Elder Care (TREC) study [37,38]. We use both computer-assisted personal interview (CAPI) and a paper survey administration in a crossover design in order to evaluate the feasibility of conducting each method and to capture time to complete and cost of each method. A vendor has developed the CAPI version of the survey [39], which is conducted by trained interviewers.

### Feasibility testing

We conducted feasibility testing to assess clarity and understanding of questions added to the TREC survey for this study. We also assessed questions where scale modifications had been made in a later version of the TREC survey, and for time to complete the survey for both CAPI and paper formats.

### Facility survey and staffing data

Facility-level data are collected from facility administrators. To collect data on facility characteristics (*e.g*., facility operation model, facility size), we are using standardized forms adapted from the TREC study [37]. We are working with facility administrators to acquire staffing data (*e.g*., sick time, absenteeism, turnover) as indicators of quality of work life. These data will be used in our regression models.

### RAI-MDS 2.0 data

Resident-level data are accessed quarterly from the RAI-MDS 2.0 databases that are maintained by data custodians. Data are received de-identified at the resident level. These data are obtained in conformity with Tri-Council Guidelines and existing health information privacy legislation in the provinces. RAI-MDS 2.0 data are used to provide feedback reports to QI teams to track their progress in making a change in resident care outcomes.

### Measures

We describe the measures in two sections: QI (process) measures and research measures.

### Quality improvement (process) measures

Process measures are collected by QI teams ongoing throughout the intervention period. Process measures include assessments of organizational (team) readiness for change, barriers to change, and a monthly QI report consisting of four measures: work group cohesion [40], work group communication [40], inter-team relationships, and team progress towards their goal. Satisfaction with the intervention will also be assessed. These measures are summarized in Table 3.

**Table 3:** Quality improvement (process) measures

Concept	Definition	Items	Reliability and Validity
Organizational readiness for change^1,2^	Facility readiness to participate in the SCOPE study.	Five items: leader support, aim and population, team membership, availability of measures, and prior experience. Teams are rated on a scale from 1 to 5 for each question and given an overall rating indicating perceived likelihood of success in the Collaborative.	Validated tool from the Institute for Healthcare Improvement (IHI).

Barriers to making a change on the unit	Perceived barriers or hindrances to making a change on the SCOPE study unit.	Six items for QI teams to complete using Yes/No responses. Five items for Senior Sponsors to complete using Yes/No responses.	Measures developed by the research team and pilot tested for face validity.

Work group cohesion^3,4^	'The degree to which an individual believes that the members of his or her work group are attracted to each other, willing to work together, and committed to the completion of the tasks and goals of the work group'^p.312^	Eight items on a seven-point Likert scale ranging from strongly disagree to strongly agree.	The original scale has demonstrated good reliability (Cronbach α = 0.92)

Work group communication^3,4^	'The degree to which information is transmitted among the members of the work group'^p.312^	Four items on a seven-point Likert scale ranging from strongly disagree to strongly agree.	The original scale has shown acceptable reliability (Cronbach α = 0.79)

Inter-team relationships^1,3^	Working relationships between the QI teams from participating facilities working on this study.	One item The rating scale ranges from 1 to 4, where 1 = no inter-team relationships 2 = starting slowly 3 = getting there 4 = strong inter-team relationships.	Validated tool from the IHI.

Team progress towards improvement goal^1,3^	Team assessment of progress in achieving their aims based on group consensus.	The rating scale ranges from 1 to 6, where 1 = team formed 2 = activity but no testing 3 = changes tested but no improvement 4 = changes tested some improvement 5 = significant improvement 6 = outstanding sustainable results.	Validated tool from the IHI.

Satisfaction with the intervention^5^	Satisfaction with participating in the QI intervention	Thirteen items	To be pilot tested during the SCOPE study.

^1 ^Adapted from Institute for Healthcare Improvement Breakthrough Series Collaborative [33] and Improvement Associates Ltd.

^2 ^http://www.improvingchroniccare.org/downloads/callgrid.doc [41]

^3 ^Completed by QI teams using a monthly tracking form

^4 ^See reference list [40].

^5 ^Adapted from Improvement Associates Ltd.

### Organizational readiness for change

Organizational (team) readiness for change is assessed by the research team's QI advisor prior to the intervention using five items adapted from IHI's collaborative readiness assessment scale [41].

### Barriers to making a change on the unit

Barriers to making a change on the unit are assessed using a scale developed by the research team based on the literature. QI team members and their senior sponsors complete these questionnaires during the intervention period.

### Monthly tracking form

Teams complete a monthly tracking form to monitor their progress towards their improvement goal and team functioning (*e.g*., work group communication).

### Satisfaction with the intervention

Satisfaction with the intervention is assessed using a thirteen item questionnaire.

### Research measures

The SCOPE survey is a minor modification of the TREC survey. The latter is composed of a suite of instruments designed in part to measure organizational context in healthcare settings, knowledge translation (*i.e*., use of research), individual factors believed to influence knowledge translation, and staff outcomes [37,38]. The Alberta Context Tool^© ^or ACT is a 51-item questionnaire within the TREC survey that measures eight dimensions of organizational context: leadership, culture, evaluation, formal interactions, informal interactions, social capital, structural resources, and organizational slack [37,38]. Reliability and validity of the ACT are reported elsewhere [37,38]. Other instruments included in the TREC survey are: self-reported knowledge translation, attitudes towards research, belief suspension, and measures of staff outcomes--burnout, health status, aggression from residents, and relationship with work [37]. Other measures added to the TREC survey for this study are empowerment (proxy measure) and quality of work life. Demographic data are also collected from study participants.

### Data quality

A research manger experienced with collecting CAPI survey data is responsible for training interviewers for a one-day session. The session is guided by a CAPI training manual and includes skills training by conducting standardized practice interviews. The instructor observes the first two interviews (using a checklist) conducted and periodic random checks thereafter to verify the standardization of the CAPI method to ensure data quality. Data cleaning and processing protocols and procedures are in place for the paper survey data for quality control. Data security and fidelity are ensured using established protocols.

### Ethical review

Ethical approval for this study was obtained from the University of Alberta, University of Calgary, and the Interior Health region of British Columbia research ethics board. We have also received operational approvals from the seven nursing homes, as well as RAI-MDS 2.0 data custodian approvals.

### Data analysis

From our previous work, we have learned that we will need at least 10 HCAs per unit for reliable aggregation statistics [42]. We will use descriptive statistics to summarize the survey data. We will use independent t-tests for pretest and posttest comparisons of mean scores on all variables. We will use a three-way analysis of variance (with random effects) to test for mean differences in the outcome variables between units, facility, and data collection time periods.

We will construct a series of regression models to assess predictors of HCA's quality of work life and use of best practices. Staff characteristics, context variables, and dose of the intervention will be the primary explanatory variables in these equations. Because of the potential for correlated responses within units and facilities, we will assess this using intra-class correlation one (ICC 1) on the response variable, and if necessary apply a cluster correction (using GEE). Scales will be assessed for their psychometric properties using standard techniques (*e.g*., factor analysis, Cronbach's alpha coefficient, item-total correlations). Resident-level RAI-MDS 2.0 data on team selected quality indicators are analyzed at the unit level using statistical process control and run charts to develop feedback reports.

An independent consultant has been contracted to complete an evaluation of the SCOPE study as a requirement from our funder [43]. We are conducting process and outcome evaluation. Examples of the evaluation questions include: What QI techniques were used by HCAs? And, what are the modifiable aspects of organizational context that are associated with successful and unsuccessful teams in the study?

## Discussion

A key challenge in the QI part of the study is facilitating sustainability of the QI intervention in this sector. In particular, two interconnected challenges we face are:

1. How can we maintain staff and leadership engagement during the study and after completion of the study?

2. How can we build improvement capability and capacity and plan for spread and sustainability of the QI work in this sector?

**Figure 3:**
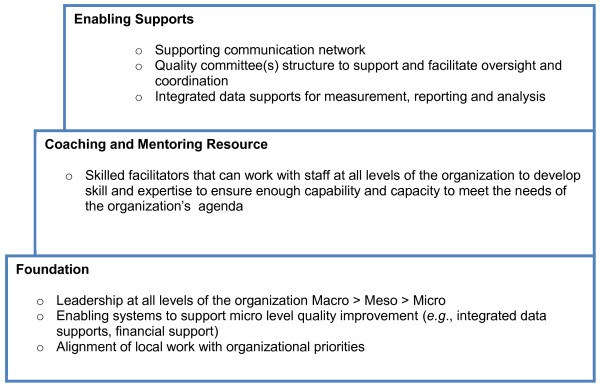
**Elements of a quality improvement infrastructure**.

Continuing success of the teams is contingent upon stability of staff. Teams could easily lose momentum and cohesion if in constant flux due to staff absenteeism and turnover. HCAs have the highest annual turnover rates in the LTC sector [18]. Sustaining QI team engagement in the study is an anticipated challenge. Managing attention is a central problem in implementation of innovation [44]. We are working with staff most of who have not been involved in QI projects or have performed at the level of a team leader. There is a steep learning curve for many staff working in a QI team that can impact staff motivation. Staff are learning new ways to implement change including: testing change through PDSAs, using baseline data for measurement, and using RAI-MDS 2.0 data to monitor progress towards their goal. Strong leadership for change, coaching, and teamwork are key strategies to the teams' success. Senior sponsor engagement and management support is crucial. In the SCOPE study, we use what are sometimes referred to as Mode II approaches to knowledge production and translation [45,46]. That is, we actively engage senior management with responsibilities for the sector and provincial quality leaders as equal partners in all aspects of the study from inception to conclusion [45,46]. Senior sponsors are involved in the learning sessions and are invited to participate in a planned closing learning congress to discuss sustainability of the intervention. Building senior sponsor and manager capability and capacity for change may foster sustainability of the QI work. The issue of spread and sustainability of interventions (knowledge use) is a critical component of knowledge translation science [47] and will require sustainability planning [48] with input from key stakeholders. QI occurs in complex adaptive systems [49]. For successful QI implementation, infrastructure needs to be considered at all levels of the organization (*i.e*., micro, meso, macro) (Figure 3).

Other challenges include limited access to resources such as computers, private space for teleconference calls, and data. For example, QI teams are asked to access their facilities' RAI-MDS 2.0 data and administrators are asked to access staffing data, both of which are infrequent requests for these groups. Time to complete study activities during scheduled work hours is another anticipated challenge. QI teams will require administrative support and coaching that will allow the necessary time to complete study activities. Thus, important factors to consider for sustainability planning include leadership support, assessment of attitudes of stakeholders, and financial implications [47].

## Conclusion

This study will result in new knowledge that is fundamental to understanding effective ways to enhance and sustain the Canadian unregulated healthcare workforce. The study methods are unique in that it combines research and QI study arms to facilitate change in the LTC sector. Acknowledging the value of investing in healthcare providers' knowledge and skills is central to improving quality in nursing homes and advancing nursing home care for older persons [50]. The SCOPE study has several potential beneficial outcomes at several levels:

1. Staff: Staff trained in QI theory, methods and techniques to improve the delivery of care and resident outcomes.

2. Residents: Improved care to the frail elderly who reside in LTC.

3. LTC sector: An empowered workforce and consequentially improvement in retention and recruitment of that workforce.

4. Provincial governments: A return on their investment in the RAI-MDS 2.0 implementation.

We plan to disseminate our findings widely targeting all relevant stakeholders including study participants, researchers, decision makers, policy makers, and senior leaders in LTC and their affiliates. We will disseminate findings and recommendations from the study such as: staff outcomes (*e.g*., burnout, job satisfaction), strategies effective in implementing QI techniques, barriers to and enablers of changing practice, and lessons learned.

## Competing interests

The authors declare that they have no competing interests.

## Authors' contributions

CAE and PGN conceived of the study and secured funding for the study, participated in the study design and coordination, and provided feedback on the draft manuscript. LAC and DB were directly involved in implementation of the intervention and data collection. GGC participated in the study design and coordination. LAC drafted the manuscript. CAE, PGN, GGC, and DB provided feedback on the draft protocol manuscript. All authors read and approved the final submitted manuscript.

## Authors' information

LAC is a Postdoctoral Fellow, Knowledge Utilization Studies Program, Faculty of Nursing, University of Alberta. LAC is supported by the Canadian Institutes of Health Research (CIHR) and Alberta Heritage Foundation for Medical Research (AHFMR) Fellowships. PGN is Professor Emeritus, Department of Family Medicine, University of Calgary. GGC is Professor, Faculty of Nursing, University of Alberta. GGC holds a CIHR New Investigator Award and an AHFMR Population Health Investigator award. DB is project manager of the SCOPE study and is a certified professional in healthcare quality. CAE is Professor, Faculty of Nursing, at the University of Alberta. CAE holds a CIHR Canada Research Chair in Knowledge Translation.
